# The Behavioral Cost of Care: Changes in Maintenance Behavior during Equine-Assisted Interventions

**DOI:** 10.3390/ani14040536

**Published:** 2024-02-06

**Authors:** Angela K. Fournier, Megan French, Elizabeth A. Letson, Joy Hanson, Thomas D. Berry, Sarah Cronin

**Affiliations:** 1 Department of Psychology, Bemidji State University, Bemidji, MN 56601, USA; sarah.cronin@bemidjistate.edu; 2 Department of Animal Welfare Science, Ethics, and Law, University of Glasgow, Glasgow G12 8QQ, UK; megan.french@glasgow.ac.uk; 3 Eagle Vista Ranch & Wellness Center, Bemidji, MN 56601, USA; elizabeth.letson@bemidjistate.edu (E.A.L.); hanson1942@tutanota.com (J.H.); 4 Department of Psychology, Christopher Newport University, Newport News, VA 23606, USA; berry@cnu.edu

**Keywords:** symbiosis, human–animal interaction, animal-assisted intervention, equine-assisted learning, anthrozooethogram, animal welfare

## Abstract

**Simple Summary:**

Psychotherapy, learning, and other interventions incorporating animals are on the rise. Researchers are interested in outcomes for human recipients and intervention animals. This study addressed the welfare of intervention animals by observing maintenance behaviors, which are daily activities that animals emit for survival (e.g., eating, moving, and sleeping). The researchers scanned the pasture before, during, and after equine-assisted (psychosocial) learning sessions, recording horses’ alertness, eating, and movement, which differed. The researchers also measured human–horse interaction, recording when humans approached the horses or the horses approached humans during equine-assisted learning sessions. These interactions were mostly from humans toward horses. Humans initiated and reciprocated interactions with horses, while horses mostly ignored or avoided interactions with humans. Applying a symbiosis framework to examine the costs and benefits of equine-assisted intervention, these findings on maintenance behavior suggest a potential cost for the animals—an interruption of or alteration in their maintenance behavior.

**Abstract:**

This study examined human–animal symbiosis in an animal-assisted intervention through observations of animal maintenance behaviors. The rise of psychotherapy, learning, and recreation incorporating animals warrants exploration of the welfare of the animals involved in these interventions. The analysis of welfare in multispecies engagements can be discussed in terms of symbiosis. Regarding an intervention’s animal provider (e.g., therapy horse) and human recipient (psychotherapy client), the balance of cost and benefit is important. Research describing human and animal *interactive behavior* during interventions is limited, whether focusing on client outcomes or animal welfare. The present study adapted ethological methods to study humans and animals in an equine-assisted intervention, observing equine maintenance behaviors and equid–human interactive behavior. Maintenance behaviors were recorded before, during, and after equine-assisted (psychosocial) learning sessions with youth, providing 1600 observations. Equine alertness, eating behavior, and ambulation varied significantly before, during, and after the equine-assisted sessions. Such interruptions of typical behavior are an important aspect of welfare and unit of analysis when examining symbiotic relationships. A total of 267 sequences of equid–human approach–response behavior were also recorded, indicating that human–animal interaction was predominantly from humans toward equids. Equids’ dominant response to human approach was no response, followed by avoidance, while humans’ dominant response to equid approach was reciprocation. The findings are discussed in terms of symbiosis and animal welfare.

## 1. Introduction

Equine-assisted services include interventions for therapy, learning, or horsemanship [1]. Psychotherapy and learning are two specific interventions incorporating equids, hereafter referred to as equine-assisted interventions. Practitioners partner with equids when serving a variety of populations, including children, veterans, and older adults, and when treating a variety of presenting concerns, including anxiety, depression, trauma, and life stressors, as well as severe and persistent mental illness [2,3]. The growing evidence base suggests promising outcomes for human recipients, including improvements in emotional awareness and regulation for underserved youth [4], improved stress management [5], decreased compassion fatigue and burnout in community care professionals [6], and improved resilience in high school students [7]. With the popularity of animal-assisted interventions on the rise, practitioners and researchers are calling for more attention to the welfare of animals working in the field. Fine and Anderson [8] include animal welfare as a critical issue facing the animal-assisted intervention field. On this topic, Peralta [9] states that engaging in animal-assisted interventions benefits the humans involved more than the animals, hinting at a human–animal cost–benefit comparison.

### 1.1. Symbiosis 

Symbiosis can be defined as an association between two or more different species [10]. The term was originally coined in 1879 by Anton de Bary [11] to mean dissimilarly named organisms living together. Martin and Schwab [12] discuss different interpretations of the term over time. Some apply a narrow definition, insisting that symbiosis only includes intimate, relatively permanent associations between species (e.g., [13,14]). Others suggest that the term be used more broadly to include less intimate and more temporary associations (e.g., [15]), for example the brief association between a hummingbird and a flower during pollination. Symbiosis is commonly discussed in terms of three subdivisions—mutualism, in which both species benefit; commensalism, in which one benefits and another is neither benefitted nor harmed; and parasitism, wherein one species benefits at the expense of another [10]. Gorman [16] suggests symbiosis be applied as an analytical framework to the domain of health and welfare, allowing for an evaluation of transactional relationships. As an example, Gorman describes *Animal Care Farming*, the provision of farming-related activities for individuals with a defined need, in terms of symbiosis, reviewing costs and benefits for human clients and farm animals. The present study applies this symbiosis framework to human and animal behavior within the equine-assisted intervention field, specifically focusing on equine *maintenance behaviors*. 

### 1.2. Maintenance Behaviors and Therapy-Animal Welfare

Peralta and Fine [17] apply the Five Domains model of animal welfare to animals working in animal-assisted interventions. The model suggests an animal’s welfare can be evaluated via the animal’s mental state, as well as four physical domains—nutrition, environment, health, and behavior [18,19,20]. For a review of the model’s development and evolution, see Mellor et al. [21]. In brief, the model currently suggests that welfare should address positive experiences and a life worth living [22], not simply a life without physical or emotional pain. In examining the behavioral domain, King and colleagues [23] observed signs of stress in dogs following an animal-assisted therapy session, including panting and yawning. Ekholm Fry [24] discusses welfare considerations for equids involved in human service work, identifying equid behaviors and human misinterpretation of equid behavior as important factors. Stress behaviors in equids can include muscle tension, an unusual head position, and tail movement [25,26]. In addition to stress-related signals, animals can show changes in maintenance behavior as a sign of disruption or distress. Maintenance behaviors are daily activities an animal emits for basic survival [27,28,29]. These include body posture; gustation, hydration, and elimination; movement; and rest. Ng et al. [30] employed an ethogram to record the maintenance behaviors in therapy dogs, noting variations in standing and ambulation between therapeutic settings and home environments. The present study extends this line of inquiry to equids, aiming to evaluate the impact of equine-assisted learning interventions on equine maintenance behaviors. Equine maintenance behaviors include ingestion, elimination, locomotion, rest, shelter seeking, and comfort-seeking behavior [27]. Regarding the symbiosis of equine-assisted interventions, the disruption of equine maintenance behavior may be an important cost of the work. According to King et al. [31], equine maintenance behaviors are influenced by environmental factors such as ambient temperature, seasonal changes, the availability of forage, and the presence of insects or other pests. Additionally, the presence of and interaction with humans may influence equine maintenance behavior.

### 1.3. Human–Animal Interaction and the Anthrozooethogram

Human–animal interaction (HAI) is an umbrella term for any interactive behaviors that occur between humans and other animals [32]. The concept is studied in a variety of contexts, such as pet ownership, entertainment, recreation, and healthcare interventions, with research progressing significantly in the last decade [33]. In efforts to better understand animal-assisted intervention processes, we (the authors) have measured reported HAI following interventions in prison [34] and in private practice [35]. In research investigating equine-assisted intervention, participants reported on the human–animal interaction scale [36] the extent to which they experienced specific interactive behaviors. Participants reported initiating interaction with (i.e., approaching) the equids “a great deal”, including petting, grooming, and spending time near them; they reported that the equids initiated interaction with them “a little” [37]. In the same study, participants reported the extent to which they avoided the equids or the equids avoided them, rating the equids as more avoidant. In a follow-up study utilizing behavioral observations, approach behavior (i.e., walking up to or moving toward another organism) was identified as a key HAI during equine-assisted learning sessions with college students [38]. Observers recorded humans approaching equids and equids approaching humans, with the former making up most interactions.

Wijnen [39] recommends interpreting therapy-animal welfare using context-specific ethograms, examining animal behavior in the context of a human’s presence or behavior. This was the second aim of the present research—to use an ethogram to study the behavioral interactions between humans and animals, hereafter referred to as an *anthrozooethogram* (the term is a combination of *anthrozoology*—the scholarly study of interactions between humans and other animals—and *ethogram*—a catalogue of natural behaviors of an animal species). An anthrozooethogram (AZE) is an HAI-specific ethogram, a resource for cataloging the behaviors of humans and animals during HAI. Based on the above-cited research, the AZE in the present study focused on one HAI—approach behavior between humans and equids. The AZE is different from other tools used to measure animal behavior, because it includes interactive behaviors between humans and animals. While it can be used to gather data relevant to equine welfare, such as it was used here, its utility is broader than that. Unlike existing models for assessing the adverse effects of interventions on equids (e.g., [40]), the AZE could also be used in investigations of mechanisms of change for interventions or just pure descriptions of human–equid interactions. 

### 1.4. The Present Study

The present study applied human–animal symbiosis as a framework for evaluating animal welfare in equine-assisted interventions. We understand this is a nontraditional application of the concept of symbiosis, but our use of the term is following others in the animal-assisted field who suggest that thinking in terms of symbiosis (i.e., [16]) may help to promote science on interventions that benefit both humans and animals. Data were collected on the maintenance behaviors of equids before, during, and after equine-assisted intervention sessions. We proposed three hypotheses given the above review of HAI research: we hypothesized that (H_1_) there would be no differences in equid maintenance behavior between the three phases (i.e., before, during, and after equine-assisted intervention sessions). Given that maintenance behaviors may be influenced by environmental factors such as HAI, we recorded human–equid *interactions* during sessions. Based on previous research showing a tendency for HAIs during interventions to be more human-initiated, we hypothesized (H_2_) that the proportion of human→equid approaches would be greater than the proportion of equid→human approaches. Finally, we predicted (H_3_) that avoidance would be a more common response to approach for human→equid approaches than equid→human approaches.

## 2. Materials and Methods

The procedures were reviewed and approved by the university’s institutional review board (IRB) regarding human subjects (approved 18 March 2022) and the university’s institutional animal care and use committee (IACUC) regarding animal subjects (approved 2 May 2022).

### 2.1. Setting 

The study took place at a mental health private practice located in the rural midwestern United States. Sessions took place and data were collected in an outdoor 10-acre pasture. The pasture is grass-covered, smooth-wire-fenced, and surrounded by pine trees. The animals had access to their usual water source—an automatic waterer—and usual supplement—two salt licks placed in the pasture. The ambient temperature during data collection ranged from 66 °F to 87 °F (*M* = 72 °F).

### 2.2. Subjects 

#### 2.2.1. Animal Subjects

The animal subjects were seven equids—six horses and one donkey—who live and work at the private practice. Table 1 provides the demographic information for the animal subjects. The equids were all adults in middle-to-older adulthood, ranging in age from 18 to 30+ years. The herd was a combination of mares and geldings which varied in breed, size, and coloring. The animals’ length of service at the private practice prior to the study ranged from 2 to 10 years (*M* = 5.54). All the animals were in good health and current on vaccinations at the time of the study. 

#### 2.2.2. Human Subjects

A total of 33 children attended sessions and participated in the study. The children were between the ages of seven and twelve years old, attended summer programming at the local Boys and Girls Club, chose to attend equine-assisted learning sessions, assented to participate in the research, and their parents consented to their participation. The children and animals were not familiar with each other; each week, a new group of children were introduced to the animals. Findings on the children’s outcomes have been reported separately (findings on the children’s outcomes showed significant correlations between human–animal interaction and resilience) [41]; this paper focuses on the animal subjects. 

### 2.3. Materials 

#### 2.3.1. Maintenance Behavior Ethogram (MBE)

Table 2 shows the ethogram designed to assist with recording maintenance behavior. The table is organized like ethograms used in previous research [28,30], with three categories of maintenance behavior—*alertness*, *postural state*, and *eating behavior*. The MBE data were collected using the Behayve application for mobile devices [42].

#### 2.3.2. Behayve Application

Behayve is a mobile application designed to collect animal behavior data in the field. Data can be recorded live, via focal sampling, scan sampling, or by recording behavior ad libitum [42]. By design, the researcher configures each study separately, defining individual subjects (e.g., Equid A) and behaviors (e.g., walking, eating) to record. The Behayve app was used on two mobile devices—an iPhone 11 and an iPad 10.2 7th Generation, both operating on the most current software version at the time—iOS 15.7.

#### 2.3.3. Approach–Response Anthrozooethogram (ARA)

Table 3 shows the ARA used to record approach behavior. The ARA is limited to a single *approach* behavior and several possible *response* behaviors. The response behaviors included *avoid*, *no response*, and *reciprocate*. The ARA data were hand recorded on an 8½ × 11-inch paper grid in landscape orientation. Each row of the grid was for recording one approach–response sequence. The grid included three columns, one for each variable recorded—the equid(s) involved, type of approach (i.e., human→equid or equid→human), and response (i.e., avoid, no response, reciprocate). For response behavior, only the ultimate outcome was recorded. For example, if an approach was met with a brief look toward the approacher (i.e., reciprocate) and then ultimately walked away (i.e., avoid), the response was recorded as “avoid”. While there are many other variables one could measure related to approach behavior (e.g., eye contact and body posture), and that have been recorded in more laboratory-based research [43], this field study employing the AZE during a client-led intervention focused on macro-level data. 

### 2.4. Procedure

#### 2.4.1. Equine-Assisted Intervention

The intervention (the researchers were not involved in planning or facilitating the intervention) consisted of group equine-assisted learning sessions. Equine-assisted learning has been defined as non-therapy experiential learning that takes place in an equine environment in the presence of horses or other equids [1]. Equine-assisted learning sessions were held twice weekly from 10:30 a.m.–12:30 p.m. There was a total of 12 sessions across 7 weeks. Each week, a different group of five to eight children would attend two sessions focused on developing social-emotional skills related to hope and resilience. The sessions were standardized, using a two-session sequence of content that was repeated each week. The sessions were planned and facilitated by a professional team—a licensed professional clinical counselor and an equine specialist. The session format began in the office with 30 min of learning social-emotional skills. Then, following a brief discussion of safety, the children spent 60 min in the pasture with the equids. All of the children in the group were in the pasture while all of the equids were at liberty (i.e., not haltered or tethered). Facilitation followed the Eagala model, a ground-based method, focusing on horses’ natural behavior, their interactions with human clients, and the clients’ response to and interpretation of the experience [44]. Eagala sessions are conducted with an equine specialist who monitors the horses’ behavior and a mental health professional who assists clients in their process of self-reflection during sessions [45,46]. The process is client-led and the equids are at liberty, free to move about the space and engage in or decline interaction with humans. The 60 min in the pasture was non-scripted and child-led. The facilitators invited the children to “spend time with the horses”, refraining from giving directives. The facilitators were present to observe the children and equids, monitor for dangerous or harmful behavior, and help the children if asked. The children decided individually if and how they would engage with the equids. If they were interested in interacting with a particular equid, they would approach the equid. The equids were free to engage with the children or to walk away. The equids’ natural behavior and responses to the children’s behavior provided an opportunity to practice the social-emotional skills discussed in the office. Interactions included the children observing, talking with, brushing, petting, haltering, and/or leading the equids. Although haltering and leading is one of the activities listed, the horses were allowed to walk away from someone trying to halter them and never forced. After 60 min, the facilitators and children went back to the office for 30 min. They discussed the experience, incorporating social-emotional skills where appropriate. 

#### 2.4.2. Data Collection

Data were collected by two researchers recording live in the pasture for two hours—30 min before the session, throughout the 60-min session, and 30 min after the session. With no other people or activities in the pasture besides the observers, the 30 min before sessions allowed for the collection of baseline behavior and the 30 min after sessions allowed for the collection of post-session behavior. Observations were overt; the observers were visible to the humans and the animals but maintained a distance of at least 20 feet from the children, facilitators, and animals. The observers moved about the pasture as needed, to maintain distance between them and the subjects, as well as to ensure a clear view of behavior. The researchers gathered data using two sampling methods concurrently—scan sampling and sequence sampling. 

#### 2.4.3. Scan Sampling of Maintenance Behavior

The researchers scan sampled the equid maintenance behavior before, during, and after the equine-assisted learning sessions. Scan sampling involves recording the current activity of a group of individuals at preselected moments in time [39]. Two independent observers scanned the pasture every five minutes, recording the behavior of each of the animals present on the MBE with the Behayve app. Each day of data collection, the seven equids were randomly split between the two observers, with one equid randomly selected to be observed by both, providing interrater reliability data. The observers recorded data on the shared equid first, so that they would be recording the same animals’ behavior at the same time.

#### 2.4.4. Sequence Sampling of Approach Behavior

Sequence sampling is for gathering data on an interactive sequence, recording behavior throughout an interaction between two or more individuals [47]. The interactive sequence observed was HAI, specifically approach–response behavior. The observers recorded each instance of a human approaching an equid (i.e., human→equid) or an equid approaching a human (i.e., equid→human). Sequence sampling was performed by the same observers, and at the same time as the scan sampling described above. Both observers attempted to record all sequences.

### 2.5. Statistical Analysis

The data were initially sorted in Microsoft Excel (version 2312), then analyzed using SPSS (version 28.0). Differences in maintenance behaviors between phases (i.e., before, during, and after sessions) were determined via chi square analyses. Differences in mean alertness were determined via a one-way ANOVA with a Tukey post-hoc analysis. Approach–response sequences were compared via percentages calculated. 

## 3. Results

### 3.1. Scanned Maintenance Behavior

A total of 1600 observations were recorded through scan sampling. The average number of maintenance behavior observations recorded per day was 66.88, ranging from 48 to 134. 

#### 3.1.1. Interrater Reliability

As mentioned earlier, one equid was randomly selected for each session to be observed by both observers independently, providing interrater comparisons for 26.6% of observations. Cohen’s kappa was calculated to determine the agreement between the two observers. On level of alertness, agreement between the two observers was substantial, *k* = 0.721, *p* < 0.001. Agreement on whether the equid was eating or not was also substantial, *k* = 0.631, *p* < 0.001. For postural state, agreement was moderate, *k* = 0.570, *p* < 0.001. Although the modal difference in time for recording each behavior was 0.00 s, in some cases, the two observers rated the behavior of the same subject at different times. When there was a difference in the timing of ratings, the mean difference was 8.12 s (SD = 10.20).

#### 3.1.2. Maintenance Behavior

**Figure 1:**
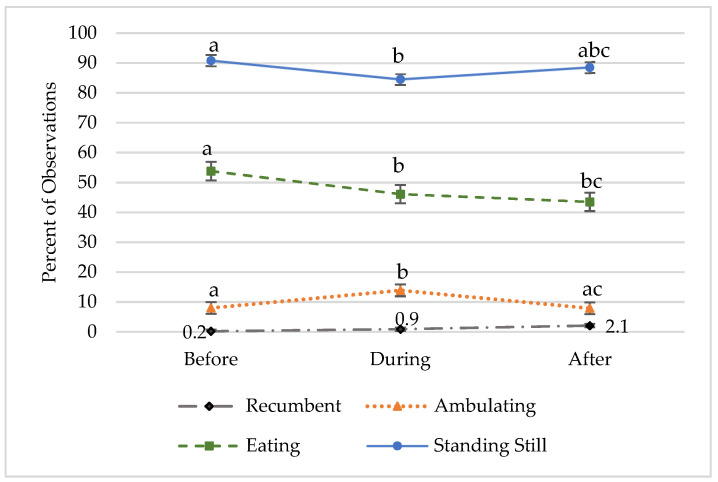
Postural state, ambulation, and eating before, during, and after equine-assisted learning sessions. Different superscript letters (a,b,c) indicate significant differences between phases, *p* < 0.01.

Overall, the equids spent most of their time standing still (86.9% of observations) and looking alert (91.2% of observations). They spent about half of their time eating (51.9% of observations). Figure 1 shows postural state, ambulation, and eating before, during, and after the equine-assisted session. The graph illustrates an increase in ambulating and a decrease in standing still during the sessions compared to before the sessions. A chi square analysis indicated that the proportion of observations where equids were standing still decreased significantly from before the session (90.8%) to during the session (84.5%); standing still increased after the session (88.5%), but not to a level significantly different from the other two phases, X^2^ (3) = 21.09, *p* = 0.002. Eating behavior followed a similar pattern. 

**Figure 2:**
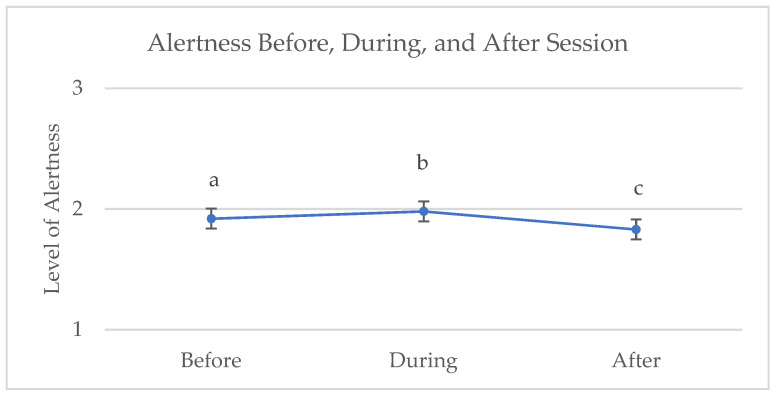
Alertness before, during, and after equine sessions. Different superscript letters (a,b,c) indicate significant differences between phases, *p* < 0.001.

The equids were eating during 53.8% of the observations before the session; this behavior decreased significantly to 46.1% of observations during the session, x^2^ (2) = 9.47, *p* = 0.009. Eating decreased slightly after the session (43.5%), but not significantly. Level of alertness was rated on a three-point scale, where 1 = sleepy, 2 = alert, and 3 = high alert. An analysis of variance indicated that alertness was significantly different at all three points in time. The mean level of alertness increased significantly from before the session (*M* = 1.92, *SD* = 0.28) to during the session (*M* = 1.98, *SD* = 0.70), and then decreased significantly after the session (*M* = 1.83, *SD* = 0.39); alertness after the session was significantly lower than before the session, *F* (2) = 25.98, *p* < 0.001. Figure 2 shows the level of alertness across phases.

### 3.2. Sequencing of HAI Behavior

#### 3.2.1. Interrater Reliability

The approach sequences were recorded by one of the two observers. For 43% of the observations, both observers recorded the same data independently. The observers agreed on 86.1% of observations on whether the approach was human→equid or equid→human and 75% for response to approach. 

#### 3.2.2. Response Overall

Table 4 provides the proportion of responses to approach. The sequence sampling revealed more human→equid sequences (80.1%, *n* = 214) than equid→human sequences (19.9%, *n* = 53). Note that response to approach differed between the equid→human and human→equid, sequences such that the humans’ most common response was reciprocation and the equids’ most common response was no response or avoidance.

## 4. Discussion

This research applied a human–animal symbiosis framework to explore the effect of equine-assisted interventions on equine welfare, suggesting a change in equine maintenance behavior as a cost in the human–animal cost–benefit comparison. We also investigated the imbricated relation between (a) equid maintenance behaviors and (b) equid–human HAI (specifically, approach–response behaviors). We make this connection as a means to evaluate the symbiotic nature between equids and humans during equine-assisted interventions. From this evaluation, we hope to better understand the fundamental patterns and processes important to equid welfare within the context of equine-assisted interventions. 

### 4.1. Changes in Maintenance Behavior 

#### 4.1.1. Increased Alertness

The scan sampling data suggested that the animals spent most of their time on their feet, alert, and eating. Overall, the equids were alert for most of the observations (91.2%), with a small proportion of observations being recorded as sleepy (7.1%) and a few instances of the equids being on high alert (1.7%). Comparing alertness across phases, the mean level of alertness increased from before the session to during the session and then decreased after the session. There are numerous factors that affect equine alertness and sleep, such as type of housing, diet, age, and time of day [48]. While we found significant changes, perhaps in response to the intervention, within a 120 min window of time, it is unknown whether the equids’ alertness differed from typical across the 24 h cycle. Gathering 24 h data periodically could allow for calculating the true average values for specific equids. This would allow within-subject comparisons, detecting changes in health and welfare over time, and determining the impact, if any, of equine-assisted intervention work on welfare. 

#### 4.1.2. Increased Ambulation, Decreased Eating

In addition to alertness, the data indicated small but significant changes in postural state and eating during the equine sessions. The equids walked more and stood still less during the sessions than before or after the sessions. Standing still, alert, and not eating were 32% of the time budget before the equine session, 36% during, and 30% after. These data suggest that the maintenance behaviors of standing still and eating may have been disrupted or altered by the equine sessions. This finding is consistent with Ng et al. [30], who found that therapy dogs also ambulated more in the therapeutic setting than at home. Houpt [29] suggests that equids spend 50–75% of their time grazing. It is unknown whether the standing still rate in our findings is an actual difference or an artifact of the scan sampling method. The observers recorded the animals’ postural state at regular intervals. The behavior of eating, as observed by researchers, involved eating grass while either standing still or slowly walking about the pasture. Because walking while eating can be quite slow, it is possible that, at the instant of recording, the animal appeared to be standing still, but if the observation lasted several more seconds, we would see the animal slowly walking and the postural state would have been recorded as ambulating. Future research could differentiate between the movement of walking from point A to point B and walking slowly while grazing. Considering the state of standing versus recumbent positions, McGreevy [49] provides time budgets of equids in various environments. For free-ranging domestic equids, approximately 80% of equids’ time is spent standing and 20% is recumbent. The present findings showed a similar pattern—the majority of the time was spent on their feet with a small percentage of time spent recumbent. 

The animals were eating for 53.8% of the observations before the equine sessions; this decreased significantly to 46.1% of the observations during the sessions and decreased further after the sessions to 43.5%. Research on equid behavior suggests that equids typically spend much of their waking hours eating. McDonnell [27] indicates that equids spend 60–80% of their time eating. A recent systematic review on equine time budgets found great variability across the literature; studies reported eating behavior budgets of 10–64% [50]. From this, it appears the equine sessions may have interrupted eating behavior, but not necessarily to abnormal levels.

In summary, the hypothesis that there would be no differences in equid maintenance behavior between the three phases (H_1_) is rejected. The scanned sampling of equid maintenance behaviors showed small but significant changes during the equine sessions, in comparison to before and after the sessions. Further research is necessary to determine whether these differences are reliable and clinically significant for animal welfare. 

### 4.2. Equid–Human HAI

The hypothesis that the proportion of human→equid approaches would be greater than the proportion of equid→human approaches (H_2_) was supported. The ratio of humans approaching to equids approaching was 80% to 20%, respectively. From an animal welfare perspective, one could assume that sessions with a lot of human approach and very little equid approach, particularly if the equid response to human approach is avoidance, might be taxing for the equid(s) or unwanted, in comparison to sessions where approach behavior is more evenly split. Perhaps an actor–receiver ratio could be calculated for equine sessions as a way of tracking HAI across animals, clients, and sessions. It could be useful to know how much human→equid HAI a herd or an individual equid is experiencing on a daily or weekly basis. Even more telling than the actor–receiver ratio could be the ratio of responses to approach behavior. 

Referring again to Table 4, when humans approached the equids, the equids often did not respond at all. For example, quite often, a child would walk up to and pet an equid, and the equid would stand there, with the same body posture, as if nothing had happened. This was observed for 61.2% of the human→equid approaches. For 24.3% of the human→equid approaches, the equid responded by avoiding, compared to 11.3% of equid→human approaches, supporting our hypothesis that avoidance would be a more common response to approach for human→equid approaches than equid→human approaches (H_3_). Avoidance behaviors included walking away or moving a part of the body away from the approaching human. There were no bolts, startles, or spooks recorded. Still, even passive avoidance can be an indication of fear, anxiety, tension, or nervousness [51]. The equids only reciprocated human approaches 14.5% of the time. On the other hand, when an equid approached a human, the human was likely to reciprocate. From an animal welfare perspective, the difference in reciprocation between equid→human (64.2%) and human→equid (14.5%) interactions may be significant. Reciprocation could be an indication of mutual benefit or at least mutual interest. Regarding the animal consenting to be part of the process, the rate of avoidance could be a helpful figure. It is noteworthy that avoidance was an option for the animals, suggesting a level of agency.

For a small proportion of sequences in which the approach was reciprocated, the researchers observed what seemed to be a *reciprocal cycle of HAI*. In these cases, an actor approaching a receiver resulted in a back-and-forth approach–reciprocate–reciprocate behavior cycle. For example, in one sequence, a human walked toward an equid, the equid turned and walked toward the human, the human reached out to pet the equid, the equid moved closer to the human and sniffed their hand, the human then began petting and talking to the equid, and the equid moved even closer to the human. More than just an approach and response, the sequence seemed to be a building of greater connection through reciprocal interactive behavior. This behavior pattern was observed in previous research, when humans interacted with dogs and, to a lesser degree, equids [38]. From the perspective of human–animal symbiosis, these sequences seemed to be consistent with Gorman’s description of mutualism [16]; both the human and equid were actors engaging in approach behavior and perhaps benefiting from the experience. Future research may examine these reciprocal cycles of HAI across species while also recording physiology or other behavioral indicators of affect. 

Returning to the concept of symbiosis [11], data from this study suggest that equid–human cost–benefit may have been imbalanced, with potential benefits to human clients occurring at the expense of the equids. The potential costs to the equids included interruption of their maintenance behaviors and human-dominated HAI. Interruption of maintenance behavior was statistically significant; further research is needed to determine the reliability and clinical significance of such interruptions. Determination of the impact must be considered in the context of other relevant variables such as an animal’s workload (e.g., number of sessions and length of sessions) and health status. Perhaps decreases in standing still and eating, such as were observed here, are harmless for an equid engaging in a small number of sessions per week, but take a toll on one doing several sessions every day. Likewise, a young healthy equid may respond less to these types of disruptions than an older equid or one with health concerns. 

### 4.3. Limitations

These findings must be considered in light of several limitations. The research was based on behavioral observations, relying on live recordings by researchers. Interrater reliability calculations indicate that observer agreement on maintenance behavior scans ranged from fair to substantial. One possible reason for disagreement among the observers was timing. The intervention was a group intervention, so there were five to eight youths in the pasture at a time and the children could interact with any of the seven equids in the herd. This number of humans and equids allowed for many behaviors and approach–response sequences to occur. The observers took scan samples every five minutes, recording each animal each time. Although the order of recording was agreed upon (e.g., first record Animal A, then Animal F, etc.), the speed at which the observers recorded could have varied, so that, at one instance, they each could have been recording two different animals, rather than the same animal, which is required for interrater agreement.

The data collection was limited to 120 min of the day and the animals’ maintenance behaviors for the remainder of the day were not recorded. It is possible that reduced eating during the intervention was compensated for later in the day, resulting in no overall difference in daily eating. Future research could incorporate 24 h observation via video recording to address this. Doing so would allow for better comparisons with 24 h time budgets in the literature. 

The data are interpreted here at an aggregate level. Further research might investigate between- and within-subject differences in maintenance behaviors to study the effects of equine-assisted interventions on individual animals. In addition, HAIs were recorded and discussed at the aggregate level. Rather than tracking an individual equid interacting with an individual human, allowing for the detection of changes in approach–response behavior from early in the session to later or from session 1 to session 2, each HAI sequence was recorded without reference to previous sequences. It may be appropriate to follow-up with a mixed method, wherein such nuances are captured with qualitative methods. 

Although we made observations of equid behavior and have discussed our findings as they may relate to the animals’ welfare, we did not include stress or discomfort indicators in our MBE. Torcivia and McDonnell [52] provide an ethogram of discomfort behaviors in equids to promote clear, universal ways to identify and communicate equine discomfort. The ethogram includes behaviors such as rolling, stomping, tail swishing, and head tossing. Perhaps ethograms should incorporate relevant discomfort behaviors when assessing equine-assisted interventions. It is important to note that equids’ outward appearance and overt behavior is just one measure of welfare. Physiological measures, such as heart rate and cortisol levels, are described as important process variables (e.g., [53]) and could provide additional information on equine welfare. Squibb et al. [54] found that equids who appeared compliant and relaxed during a challenging task showed signs of stress in physiological measures. Therefore, while overt behavior is an important indicator of equine welfare, it may not be sufficient alone. 

The data for this study were collected from one herd, and this particular herd was of qn older age. While there is no consensus on the upper age limits for therapy animals [55], age could be associated with changes in behavior, including interactive behavior. One study investigating equine age and behavior found that an increased age was associated with greater boldness (i.e., less shy or fearful) [56], a variable relevant to social interaction. Continued research with a larger, more diverse sample of equids is necessary before drawing conclusions on the scan/sequence data. The next step may be replicating this study with multiple herds, gathering data on context variables in addition to observational data. Citizen science, which is the involvement of the public in scientific research [57], may be a useful approach, as it is appropriate for research requiring many data points on variables or events that can be observed by citizens in the community. Practitioners of equine-assisted services could serve as citizen scientists, reporting on maintenance behaviors before, during, and after sessions at their practice. Animal-assisted professionals were helpful in previous research measuring HAI during interventions [37], highlighting the many AAI practitioners who are not researchers but appreciate the need for research to support their work. A similar approach could be applied to study maintenance behaviors during equine-assisted interventions, contributing to a larger database of working equids and the effect of interventions on maintenance behavior. 

## 5. Conclusions

Applying a symbiosis framework, these findings suggest that disruptions to typical behavior could be a cost of care for equids working in learning and mental health interventions. The study revealed a significant increase in equid alertness during sessions, accompanied by changes in ambulation and eating behavior. The equids walked more and ate less during sessions, suggesting potential disruptions to their maintenance behaviors. An analysis of equid–human interaction indicated a higher proportion of human approaches compared to equid approaches, with differences in response to approach as well. Humans were likely to reciprocate equid approach and equids were most likely to ignore human approach. The research suggests potential imbalances in the cost–benefit relationship, with human client benefits at the possible expense of equids, particularly in terms of interrupted maintenance behaviors and human-dominated interactions. The study calls for further research to determine the reliability and clinical significance of these findings and emphasizes the need to consider contextual variables, such as workload and health status, when assessing the impact of equine-assisted interventions on equine welfare.

## Figures and Tables

**Table 1:** Animal subject demographics.

Animal	Breed	Coloring	Sex	Age	Size	Years of Service
A	Norwegian Fjord	Brown Dun	Gelding	23	1.34 m	2.1
B	Norwegian Fjord	Brown Dun	Mare	22	1.34 m	2.1
C	Paint Cross	Black and White (Tobiano)	Gelding	30+	1.52 m	5.7
D	Paint Cross	Black and White (Tobiano)	Mare	30+	1.52 m	5.7
E	American Paint/ Quarter Horse	Red Dun and White (Tobiano)	Mare	19	1.43 m	10
F	Miniature Horse	White	Gelding	29	0.86 m	7.7
G	Miniature Donkey	Brown	Jennet	18	0.91 m	5

**Table 2:** Ethogram for recording maintenance behavior (MBE).

Behavior	Definition
**Alertness**	
Alert	Head up high, ears upright and forward, eyes wide, body rigid
Awake	Head up, ears relaxed, eyes open and relaxed
Drowsy or Asleep	Standing still or recumbent with eyes closed or almost closed
**Postural State**	
Ambulating	Walk, trot, or canter; whole body moves from one point to another
Recumbent	Body is on ground and staying on ground for several moments
Standing still	Feet on ground and staying in one place, no ambulation
**Eating**	
Eating	Head down, mouth on grass, chewing and swallowing

**Table 3:** Anthrozooethogram for recording approach–response behavior (ARA).

**Approach Behavior**	**Definition**
Approach	Walk up to another, move toward another, initiate interaction with another
**Response Behavior**	**Definition**
Avoid	Move away from someone approaching
No Response	Behavior does not change when approached
Reciprocate	Respond to approach from another by emitting approach behavior toward them, look toward or move toward approacher

**Table 4:** Approach–response behavior, comparing equid→human and human→equid sequences.

	Response % (*n*)		
Actor–Receiver	Avoidance	No Response	Reciprocation
Equid→Human (*n* = 52)	11.3% (6)	22.6% (12)	64.2% (34)
Human→Equid (*n* = 214)	24.3% (52)	61.2% (131)	14.5% (31)

## Data Availability

The raw data supporting the conclusions of this article will be made available by the authors, without undue reservation.
